# Thymosin beta 10 is a key regulator of tumorigenesis and metastasis and a novel serum marker in breast cancer

**DOI:** 10.1186/s13058-016-0785-2

**Published:** 2017-02-08

**Authors:** Xin Zhang, Dong Ren, Ling Guo, Lan Wang, Shu Wu, Chuyong Lin, Liping Ye, Jinrong Zhu, Jun Li, Libing Song, Huanxin Lin, Zhenyu He

**Affiliations:** 1 Department of Radiation Oncology, Sun Yat-sen University Cancer Center, State Key Laboratory of Oncology in South China, Collaborative Innovation Center of Cancer Medicine, Guangzhou, Guangdong 510060 People’s Republic of China; 2 Sun Yat-sen University Cancer Center, State Key Laboratory of Oncology in South China, Collaborative Innovation Center for Cancer Medicine, Guangzhou, 510060 China; 3 Department of Orthopaedic Surgery/Orthopaedic Research Institute, The First Affiliated Hospital of Sun Yat-sen University, Guangzhou, Guangdong Province 510080 China; 4 Department of Nasopharyngeal Carcinoma, Sun Yat-sen University Cancer Center, State Key Laboratory of Oncology in South China, Collaborative Innovation Center of Cancer Medicine, Guangzhou, 510060 China; 5 Department of Pathogen Biology and Immunology, School of Basic Courses, Guangdong Pharmaceutical University, Guangzhou, 510006 China; 6 Department of Biochemistry, hongshan School of Medicine, Sun Yat-sen University, Guangzhou, 510080 China

**Keywords:** TMSB10, Proliferation, Cell cycle, Tumorigenesis, Metastasis, Serum marker, AKT/FOXO signaling, Breast cancer

## Abstract

**Background:**

Thymosin beta 10 (TMSB10) has been demonstrated to be involved in the malignant process of many cancers. The purpose of this study was to determine the biological roles and clinical significance of TMSB10 in breast cancer and to identify whether TMSB10 might be used as a serum marker for the diagnosis of breast cancer.

**Methods:**

TMSB10 expression was evaluated by immunohistochemical analysis (IHC) of 253 breast tumors and ELISA of serum from 80 patients with breast cancer. Statistical analysis was performed to explore the correlation between TMSB10 expression and clinicopathological features in breast cancer. Univariate and multivariate Cox regression analysis were performed to examine the association between TMSB10 expression and overall survival and metastatic status. In vitro and in vivo assays were performed to assess the biological roles of TMSB10 in breast cancer. Western blotting and luciferase assays were examined to identify the underlying pathway involved in the tumor-promoting role of TMSB10.

**Results:**

We found TMSB10 was upregulated in breast cancer cells and tissues. Univariate and multivariate analysis demonstrated that high TMSB10 expression significantly correlated with clinicopathological features, poor prognosis and distant metastases in patients with breast cancer. Overexpression of TMSB10 promotes, while silencing of TMSB10 inhibits, proliferation, invasion and migration of breast cancer cells in vitro and in vivo. Our results further reveal that TMSB10 promotes the proliferation, invasion and migration of breast cancer cells via AKT/FOXO signaling, which is antagonized by the AKT kinase inhibitor perifosine. Importantly, the expression of TMSB10 is significantly elevated in the serum of patients with breast cancer and is positively associated with clinical stages of breast cancer.

**Conclusion:**

TMSB10 may hold promise as a minimally invasive serum cancer biomarker for the diagnosis of breast cancer and a potential therapeutic target which will facilitate the development of a novel therapeutic strategy against breast cancer.

**Electronic supplementary material:**

The online version of this article (doi:10.1186/s13058-016-0785-2) contains supplementary material, which is available to authorized users.

## Background

Breast cancer is the most frequent malignancy in women and the second leading cause of cancer-related deaths worldwide [1]. Despite great progress in the systemic treatment of tumors over recent years, tumor progression and distant metastasis are still the primary issues affecting the survival of patients with breast cancer [2]. Recent extensive research has indicated that introduction of an increasing number of individualized molecular targeted therapies into routine clinical treatment mirrors their importance in modern cancer prevention and treatment. For example, targeted therapy for hereditary breast cancer has become a reality with the approval of olaparib for breast cancer gene 1 (*BRCA*)-associated breast cancer [3]. Therefore, better understanding of potential biomarkers and therapeutic targets of breast cancer will facilitate improvement in the survival rate of patients with breast cancer.

The beta-thymosins, which were originally identified from the thymus, are subfamilies of thymosins [4]. The beta-thymosin family, primarily including thymosin beta 4 (TMSB4), TMSB10 and TMSB15, function as actin-sequestering proteins to inhibit actin polymerization and disrupt the formation of F-actin [5]. Furthermore, beta-thymosins exhibit diverse physiological functions beyond actin sequestration, including tissue development and regeneration, anti-inflammatory effects, and induction of insulin secretion [6–10]. Recent studies have focused on the biological roles of beta-thymosins in the progression and metastasis of various types of cancer. For example, TMSB4 overexpression correlates with advanced stages of cancer and shorter overall survival by regulating invasiveness and stemness in glioma cells [11]; TMSB15A is upregulated in transforming growth factor beta 1-treated breast cancer cells, and TMSB15B is involved in epidermal growth factor-induced migration of prostate cancer cells [12]. TMSB10 is generally upregulated in many cancers [13–16]. It is worth noting that Bouchal et al. report that TMSB10 is positively associated with high-grade aggressive breast cancer [17], but the underlying mechanism by which TMSB10 promotes breast cancer progression and metastasis remains unclear, which is yet to be further elucidated.

In this study, we report that TMSB10 is significantly elevated in human breast cancer cells and tissues, and correlates with advanced clinicopathological features, metastasis status and poor prognosis. Overexpression of TMSB10 promotes, while silencing of TMSB10 inhibits, proliferation, invasion and migration of breast cancer cells in vitro and in vivo. Our results further reveal that upregulating TMSB10 promoted the proliferation, migration and invasion of breast cancer cells by activating AKT/FOXO signaling. Importantly, we found that the expression level of TMSB10 in the serum of patients with breast cancer positively correlates with clinical stages of cancer in these patients. These findings indicate that TMSB10 may be used as a valuable serum biomarker for the diagnosis of breast cancer and as a potential therapeutic target for the treatment of breast cancer.

## Methods

### Vectors and retroviral infection

HumanTMSB10 cDNA was purchased form (Vigene Biosciences, Shandong, China) and cloned into the pSin-EF2 plasmid (addgene #16578, Cambridge, MA, USA). Knockdown of endogenous TMSB10 was performed by cloning two short hairpin RNA (shRNA) oligonucleotides into the pSUPER-puro-retro vector (OligoEngine, Seattle, WA, USA). The sequences of the two separate shRNA fragments are: RNAi#1: CCGGCCCAGTCGTGATGTGGAGGAACTCGAGTTCCTCCACATCACGACTGGGTTTTTG and RNAi#2: CCGGGCCGACCAAAGAGACCATTGACTCGAGTCAATGGTCTCTTTGGTCGGCTTTTTG (synthesized by Invitrogen). Retroviral production and infection were performed according to Weinberg et al. [18]. The reporter plasmid for the quantitative detection of forkhead box O (FOXO) transcriptional activity was generated using the pGL3-Enhancer plasmid (Promega, Madison, WI, USA) [19]. Cells were treated with Perifosine (KRX-0401) (Cell Signaling, Beverly, MA, USA) at the indicated final concentrations (30 μM).

### Tissue specimens and clinicopathological characteristics

The total 253 paraffin-embedded, archived invasive breast cancer samples used in this study were histopathologically and clinically diagnosed at the Sun Yat-sen University Cancer Center (Guangzhou, China) between 1995 and 2009. A total of 100 invasive samples of fresh breast cancer tissue (with 9 paired samples from adjacent normal tissue) and serum specimens were obtained from age-matched women during surgery at the Sun Yat-sen University Cancer Center between January 2015 and December 2015. Clinicopathological classification and staging were determined according to the criteria of the American Joint Committee on Cancer (AJCC) [20]. Patient consent and approval from the Sun Yat-Sen University Cancer Center Institutional Review Board (IRB) were obtained prior to the use of these clinical materials for research purposes (approval number GZR2013-024). The clinicopathological features of the patients are summarized in Additional files 1 and 2: Table S1 and Table S2.

### Statistical analysis

All statistical analyses were performed using SPSS version 19.0 (SPSS Inc., Chicago, IL, USA) [18]. Associations between TMSB10 expression and clinicopathological characteristics of the patients were analyzed using the chi-squared test. Survival curves were plotted using the Kaplan-Meier method and compared using the log-rank test. Survival data were evaluated using univariate and multivariate Cox regression analyses [21]. A two-tailed *P* value below 0.05 was considered statistically significant in all experiments.

## Results

### TMSB10 is upregulated in breast cancer cell lines and tissues

**Fig. 1:**
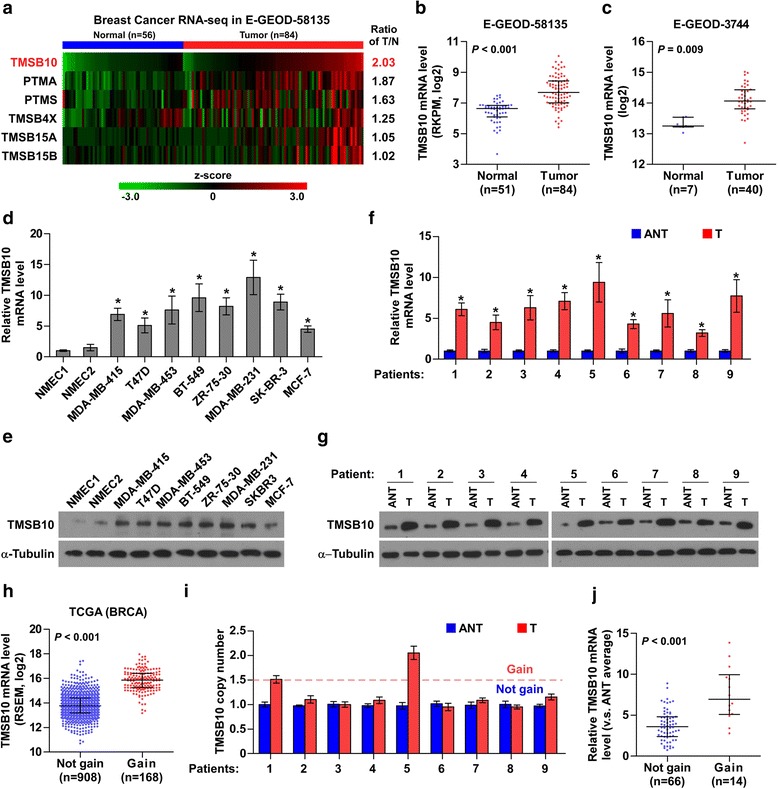
Thymosin beta 10 (*TMSB10*) is upregulated in breast cancer. **a** TMSB10, prothymosin, alpha (*PTMA*), parathymosin (*PTMS*), TMSB4X, TMSB15A and TMSB15B expression was upregulated to varying degrees in the RNA sequencing profile of breast cancer of the E-GEOD-58135 dataset. **b** and **c** Expression of TMSB10 was upregulated in breast cancer tissues and breast cancer cells compared with normal breast tissue samples and epithelium cells in the E-GEOD-58135 and E-GEOD-3744 profiles, respectively. Each *bar* represents the median values ± quartile values. **d** and **e** Real-time PCR and western blotting analysis of TMSB10 expression in NMEC1, NMEC2 and breast cancer cell lines. Glyceraldehyde-3-phosphate dehydrogenase was used as endogenous control in RT-PCR and α-Tubulin was detected as a loading control in the western blot. Each *bar* represents the mean values ± SD of three independent experiments. *P < 0.05. **f** and **g** mRNA and protein expression levels of TMSB10 in nine paired breast cancer tissue samples. The average TMSB10 mRNA expression level was normalized to the expression of GDPDH. α-Tubulin was detected as a loading control. Each *bar* represents the mean values ± SD of three independent experiments. *P < 0.05. **h** The average expression level of TMSB10 in patients with breast cancer with gains (amplification) was higher than those without gains in The Cancer Genome Atlas (*TCGA*) breast cancer dataset. Each *bar* represents the median values ± quartile values. **i** Change in copy number of TMSB10 due to gains in nine paired patients with breast cancer. Each *bar* represents the mean values ± SE. **j** Average expression level of TMSB10 in patients with breast cancer with gains was higher than those without gains in our breast cancer tissues. Each *bar* represents the median values ± quartile values. *BRCA* Breast cancer gene 1, *ANT* Adjacent normal tissue, *T* Tumor

We first analyzed expression levels of primary thymosin-associated proteins in the RNA sequencing data from E-GEOD-58135 and The Cancer Genome Atlas (TCGA) datasets, including TMSB4, TMSB10, TMSB15, prothymosin, alpha (PTMA) and parathymosin (PTMS), which have been reported to be implicated in the development and progression of different types of cancer, including breast cancer [11–13, 17, 22, 23] and found that expression levels of TMSB10, PTMS and PTMA were upregulated to varying degrees in breast cancer tissues compared with normal tissues, particularly TMSB10 at the highest level (2.03-fold and 1.45-fold change, respectively) (Fig. 1a and Additional file 3: Figure S1a).

Subsequent analysis of TMSB10 expression in breast cancer datasets revealed that TMSB10 was upregulated in breast cancer tissues and breast cancer cells compared with normal breast tissue samples and epithelium cells (Fig. 1b, c and Additional file 3: Figure S1b). To further confirm the results from public datasets, we examined the expression of TMSB10 in two normal breast epithelial cell lines and eight breast cancer cell lines by real-time PCR and western blotting, and found that mRNA and protein levels of TMSB10 were upregulated in breast cancer cells compared with NMEC1 and NMEC2 (Fig. 1d and e). Moreover, TMSB10 expression was markedly increased in nine paired breast cancer tissue samples compared with the matched adjacent normal tissues (Fig. 1f and g).

We further analyzed the mRNA expression of TMSB10 in the breast cancer tissue samples of TCGA different molecular subtypes, which contribute to the significant heterogeneity of breast cancer and found that compared with normal tissues, TMSB10 was differentially upregulated in all subtypes except the luminal A subtype, and was strikingly higher in the basal-like and human epidermal growth factor receptor 2 (Her2)-enriched subtypes (Additional file 3: Figure S1c and d). Taken together, these results indicate that TMSB10 may be involved in human breast cancer progression.

We further investigated the specific mechanism underlying the overexpression of TMSB10 in breast cancer. Through analyzing the methylation array dataset of breast cancer from TCGA, we found that in the methylation levels of TMSB10 there was no obvious discrepancy between tumor and the matched adjacent normal tissue (Additional file 3: Figure S1e). On analysis of breast cancer datasets from TCGA and Tumorscape, recurrent gains (amplification) were found in approximately 15% of patients with breast cancer (Additional file 3: Figure S1f and g). The average expression level of TMSB10 in patients with breast cancer with gains was dramatically higher than those without gains (Fig. 1h) (Additional files 4, 5 and 6). We further examined the TMSB10 in our nine paired breast cancer tissue samples using RT-PCR and identified amplification in two of nine breast cancer tissue samples (Fig. 1i). The mRNA expression level of TMSB10 in patients with breast cancer with gains was significantly elevated compared those without gains (Fig. 1j). These results indicate that recurrent gains are involved in the TMSB10 overexpression in breast cancer.

### Upregulation of TMSB10 correlates with advanced clinicopathological features and poor prognosis in breast cancer

**Fig. 2:**
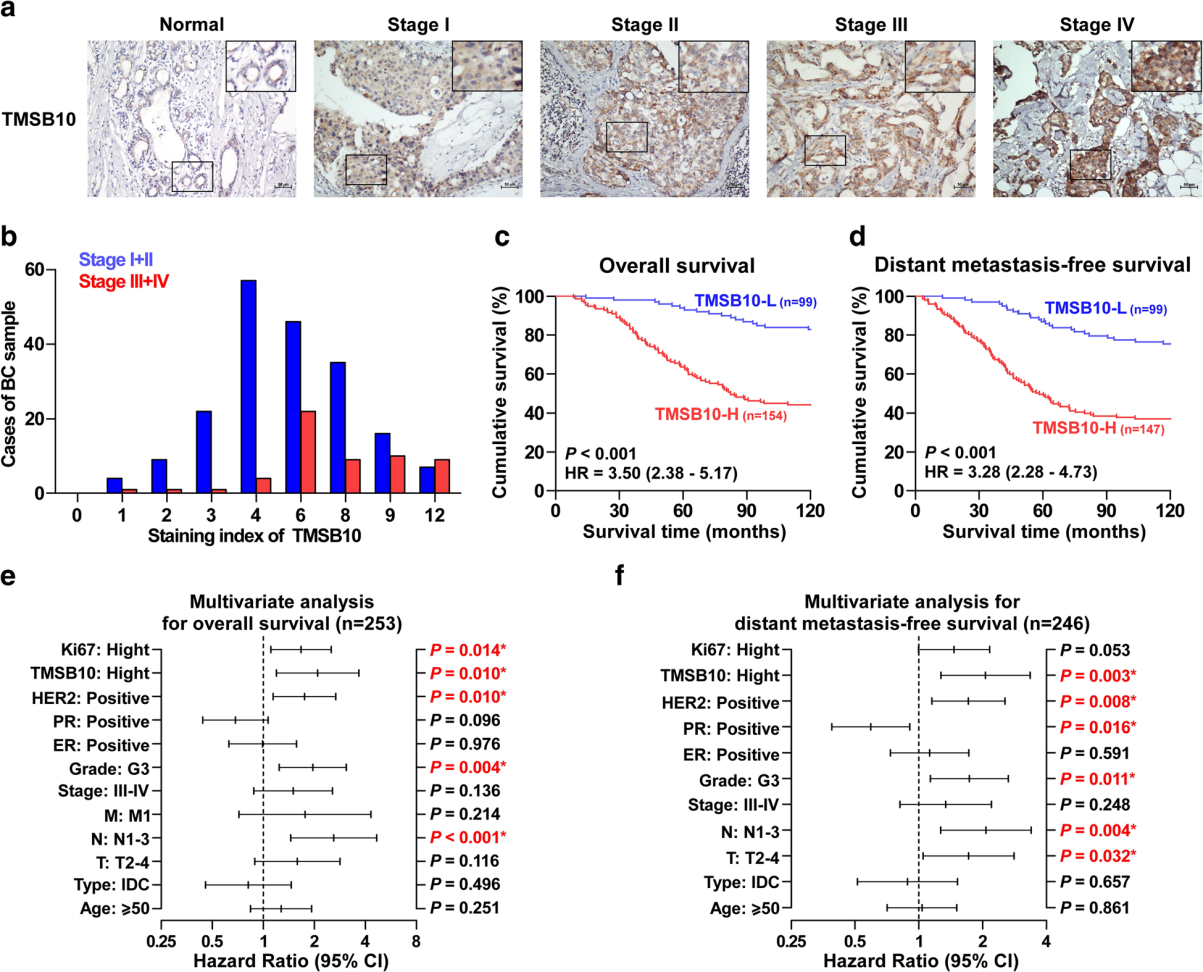
High expression of thymosin beta 10 (*TMSB10*) in breast cancer tissue samples correlates with advanced clinicopathological features and poor patient survival. **a** Representative images of TMSB10 expression in normal breast tissue and breast cancer tissue samples at different clinical stages. **b** Number of breast cancer tissue samples at stages I-II or III-IV in different staining index groups on immunohistochemical analysis (IHC). **c** and **d** Kaplan-Meier overall survival and distant metastasis-free survival curves for patients with breast cancer stratified by high and low expression of TMSB10. **e** and **f** Multivariate Cox regression analysis to evaluate the significance of the association between TMSB10 expression and overall survival and metastasis-free survival. Hazard ratios are presented by log2 transformation. *PR* progesterone receptor, *ER* estrogen receptor, *T* tumor, *N* nodes *M* metastasis

We further examined TMSB10 expression by immunohistochemical analysis of 253 human breast cancer tissue samples (Additional file 1: Table S1) and found TMSB10 expression was primarily detected within the cytoplasm and the expression levels of TMSB10 positively correlated with clinical stages (Fig. 2a and b). High expression of TMSB10 was observed in 154/253 breast cancer tissue samples (60.9%) (Additional file 7: Figure S2a). In addition, there were strong positive associations between TMSB10 expression and clinical stage, tumor (T) classification, node (N) classification, metastasis (M) classification, pathological grade, estrogen receptor (ER) status and progesterone receptor (PR) status (Additional file 8: Table S3). Kaplan-Meier survival analysis revealed that patients with high TMSB10 expression had shorter overall survival (*P* < 0.001; hazard ratio = 3.50, 95% CI = 2.38 to 5.17; Fig. 2c) and distant metastasis-free survival (*P* < 0.001; hazard ratio = 3.28, 95% CI = 2.28 to 4.73; Fig. 2d), which were consistent with TCGA and Kaplan-Meier Plotter (BC) profiles (Additional file 7: Figure S2b-f). Univariate Cox regression analysis indicated that patients with high TMSB10 expression had shorter overall survival (*P* < 0.001; hazard ratio = 4.62, 95% CI = 2.75 to 7.79) and metastasis-free survival (*P* < 0.001; hazard ratio = 3.89, 95% CI = 2.48 to 6.11) compared to patients with low TMSB10 expression (Additional file 9 and 10: Table S4 and S5).

Multivariate Cox regression analysis suggested that TMSB10 may be an independent factor for predicting poor survival (*P* = 0.010; hazard ratio = 2.10, 95% CI = 1.20 to 3.67; Fig. 2e) and distant metastasis status (P = 0.003; hazard ratio = 2.07, 95% CI = 1.27 to 3.35; Fig. 2f). Furthermore, the analysis of breast cancer datasets from Kaplan-Meier Plotter revealed that patients with high TMSB10 expression exhibited longer disease-free survival compared to patients with low TMSB10 expression after chemotherapy, but not seen in patients treated with endocrine therapy or without systemic treatment (Additional file 7: Figure S2g-i), indicating that TMSB10 may be used as a potential indicator of chemotherapeutic response to stratify patients for the treatment of breast cancer. Collectively, these results demonstrate that the overexpression of TMSB10 correlates with poor prognosis and distant metastatic status in patients with breast cancer.

### TMSB10 promotes the proliferation and tumorigenesis of breast cancer

**Fig. 3:**
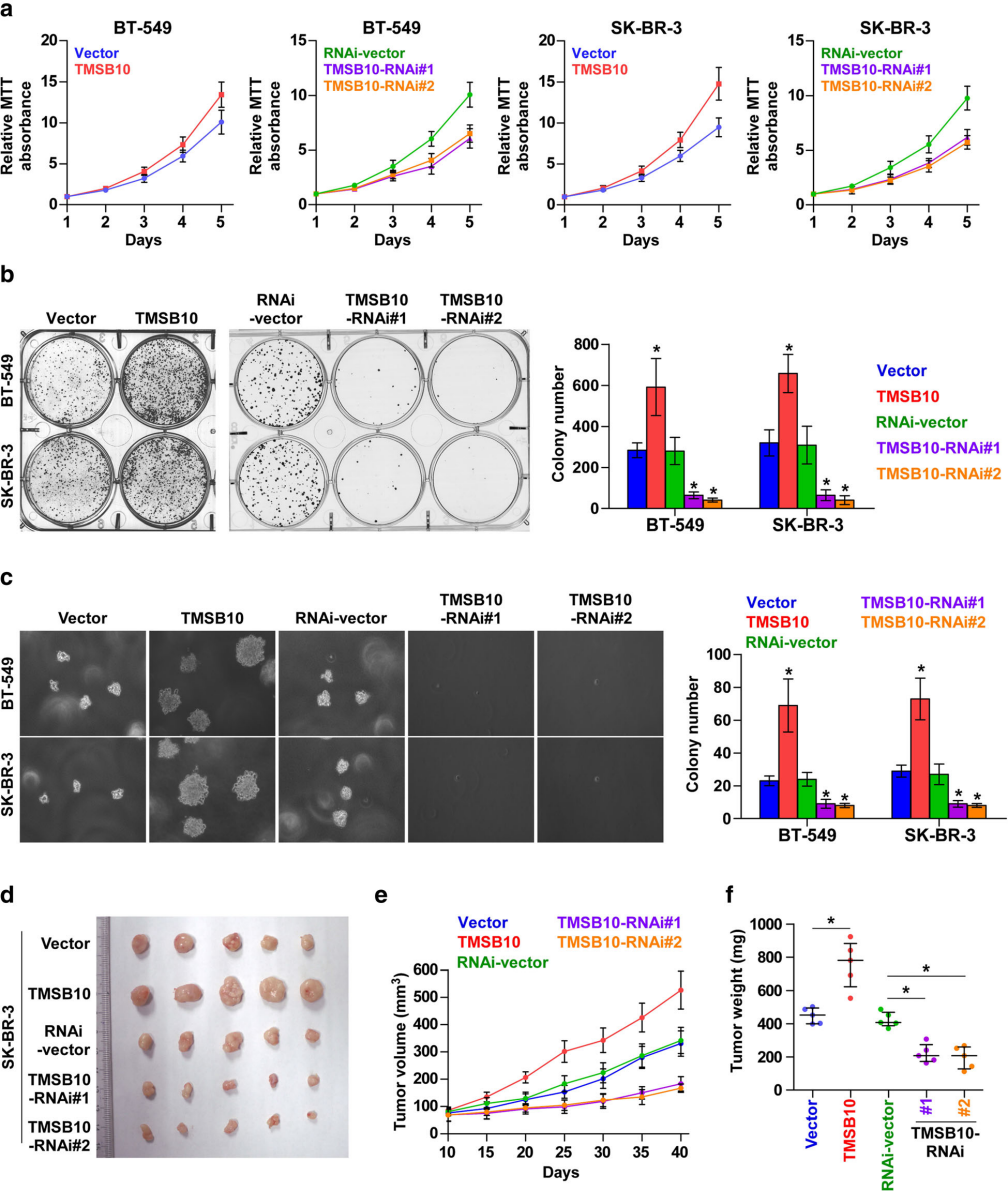
Thymosin beta 10 (*TMSB10*) promotes the proliferation and tumorigenesis of breast cancer cells in vitro and in vivo. **a** In MTT assays, overexpression of TMSB10 significantly increased the growth rate of the indicated cells, while downregulation of endogenous TMSB10 significantly reduced the growth rate. Each *bar* represents the mean values ± SD of three independent experiments. **b** Overexpression of TMSB10 increased, while downregulation of endogenous TMSB10 reduced, the mean colony number in the colony formation assay. Each *bar* represents the mean values ± SD of three independent experiments. *P < 0.05. **c** Representative micrographs and colony numbers in the anchorage-independent growth assay. Each *bar* represents the mean values ± SD of three independent experiments. *P < 0.05. **d** Images of excised tumors from five BALB/c mice at 30 days after injection with the indicated cells. **e** Tumor volumes were measured every 5 days. Each *bar* represents the median values ± quartile values. **f** Average weight of excised tumors from the indicated mice. Each *bar* represents the median values ± quartile values. *P < 0.05

To determine the biological roles of TMSB10 in breast cancer, we constructed TMSB10 stably expressing BT-549 and SK-BR-3 breast cancer cell lines by ectopically overexpressing TMSB10 and endogenously knocking down TMSB10 by retrovirus infection. Real-time PCR and western blot were performed to measure the mRNA and protein levels of TMSB10 expression (Additional file 11: Figure S3a and b). MTT assays revealed that ectopic expression of TMSB10 significantly increased, while silencing of TMSB10 reduced, the cell numbers in breast cancer cells (Fig. 3a). Colony formation assay indicated that the upregulation of TMSB10 enhanced the colony-forming abilities of breast cells. Conversely, downregulation of TMSB10 decreased the colony-forming ability (Fig. 3b).

To investigate the effects of TMSB10 on the tumorigenic activity of breast cancer cells, we performed an anchorage-independent growth assay and found overexpression of TMSB10 enhanced, while silencing of TMSB10 reduced, the anchorage-independent growth ability of breast cancer cells (Fig. 3c). We next evaluated the effect of TMSB10 on tumorigenesis in vivo. As shown in Fig. 3d-f, tumor volumes and weight were increased significantly in the TMSB10-overexpression group compared with the control group. In contrast, the tumors formed by the TMSB10-silenced cells were smaller, with lower tumor volume and weight compared to the control group. On IHC the TMSB10 overexpressing tumor tissues had higher Ki67 proliferation indexes, whereas the TMSB10-silenced tumor tissues had reduced numbers of Ki67-positive cells (Additional file 11: Figure S3c), which is consistent with the results of analysis of clinical correlation between TMSB10 and Ki67 (Additional file 8: Table S3). Taken together, these results suggest that TMSB10 promotes the tumorigenicity of breast cancer cells by modulating proliferation in breast cancer.

We next explored the specific mechanism underlying the pro-proliferative role of TMSB10 in breast cancer cells. The bromodeoxyuridine (BrdU) incorporation assay demonstrated that the percentages of cells with incorporated BrdU were increased in TMSB10-overexpressing cells, but reduced in TMSB10-silenced cells (Additional file 11: Figure S3d). Moreover, real-time PCR and western blotting analysis revealed that cyclinD1, cyclinE1 and p-Rb were upregulated in TMSB10-overexpressing cells but decreased in TMSB10-silenced cells. Conversely, the cell cycle inhibitors p21Cip1 and p27Kip1 were decreased in TMSB10-overexpressing cells but increased in TMSB10-silenced cells (Additional file 11: Figure S3e and S3f). Collectively, these results indicate that TMSB10 promotes proliferation and tumorigenesis of breast cancer cells by accelerating the cell cycle.

### TMSB10 promotes metastasis of breast cancer cells in vitro and in vivo

**Fig. 4:**
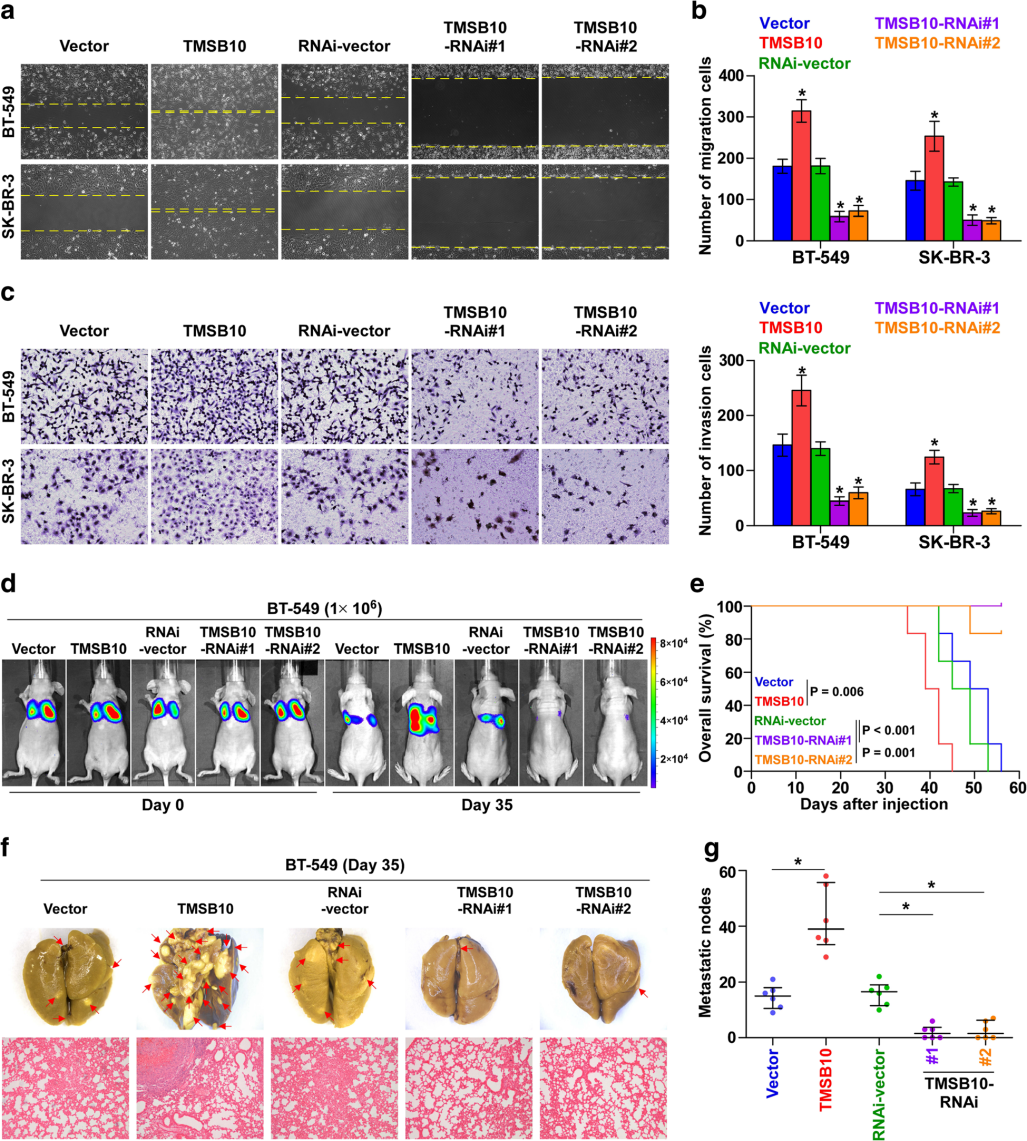
Thymosin beta 10 (*TMSB10*) promotes lung metastasis in vitro and in vivo. **a** In wound healing assays, overexpression of TMSB10 significantly increased the migration ability of the indicated cells, while downregulation of endogenous TMSB10 reduced the migration ability of the indicated cells. **b** Overexpression of TMSB10 increased, while downregulation of TMSB10 reduced, the migration ability of the indicated cells in the migration assay. Each *bar* represents the mean values ± SD of three independent experiments. *P < 0.05. **c** Overexpression of TMSB10 increased, while downregulation of TMSB10 reduced, the invasive ability of the indicated cells in the invasion assay. Each *bar* represents the mean values ± SD of three independent experiments. *P < 0.05. **d** In vivo metastasis assays of BT-549 cells with TMSB10 overexpression or knockdown. Bioluminescent imaging (BLI) was used to weekly monitor the lung metastasis burden of xenografted animals. Shown are BLI images of representative mice on days 0 and 35 after injection, respectively. The *color scale* depicts the photon flux (photons per second) emitted from the metastatic cells. **e** Kaplan-Meier survival curves for mice. **f** and **g** Lung metastases in mice were confirmed by H&E staining. *Arrows* indicate the metastatic colonization of tumor cells (*left panel*). The numbers of lung tumor nests in each group was counted under a low power field and are presented as the median values ± quartile values (*right panel*). *P < 0.05

On gene set enrichment analysis (GSEA) based on data on mRNA expression of TMSB10 from the TCGA, high expression of TMSB10 significantly correlated with metastasis-associated gene signatures (Additional file 12: Figure S4a). To investigate the effects of TMSB10 on the metastatic ability of breast cancer cells, we performed cell wound healing and migration assay and found overexpression of TMSB10 enhanced, while silencing of TMSB10 reduced, the migration ability of breast cancer cells (Fig. 4a and b). Cell invasion and 3D spheroid invasion assay demonstrated that upregulating TMSB10 increased the invasive ability of breast cancer cells with typical highly aggressive and invasive cell morphology, which presented a greater number of outward projections (Fig. 4c and Additional file 12: Figure S4b). However, silencing TMSB10 displayed the opposite effect on the invasive ability of breast cancer cells. We further examined the effects of TMSB10 on breast cancer metastasis in lung colonization models. TMSB10-overexpressing and vector-transduced BT-549 cells were injected into the BALB/c nude mice via the lateral tail veins respectively and metastatic activity was examined by bioluminescence imaging (BLI) of luciferase-transduced cells and H&E staining of lung metastasis sections. We observed that upregulating TMSB10 significantly enhanced the lung metastasis burden of BT-549 and shortened the survival of mice (Fig. 4d-g). In contrast, silencing TMSB10 attenuated the development of lung metastasis and increased the survival of mice (Fig. 4d-g). Collectively, these results indicate that TMSB10 promotes the metastasis capability of breast cancer cells.

### AKT/FOXO signaling is essential for the tumor-promoting role of TMSB10 in breast cancer

**Fig. 5:**
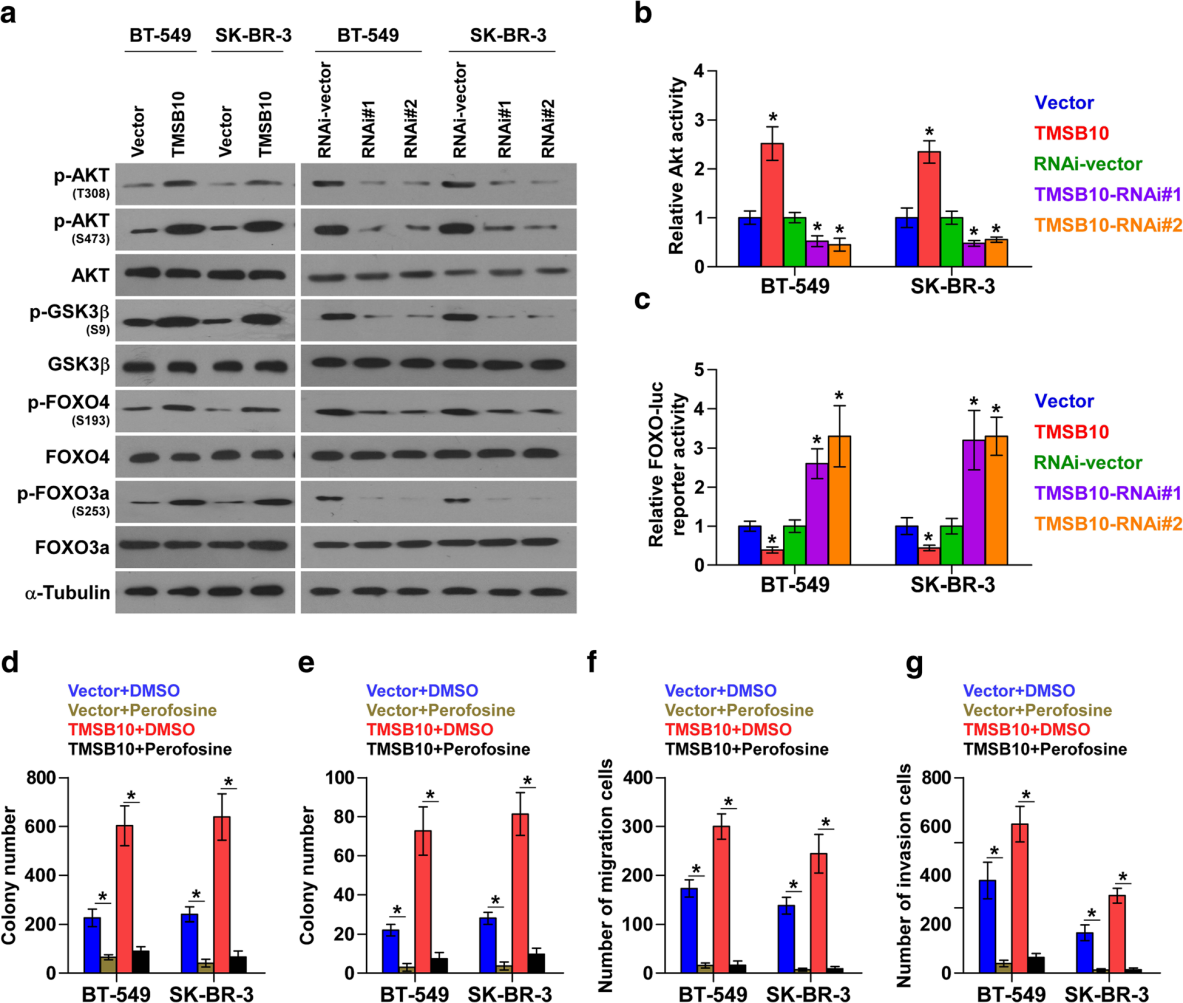
Thymosin beta 10 (*TMSB10*) promotes proliferation and metastasis via activating the AKT/FOXO signaling pathway in breast cancer. **a** Western blotting analysis of AKT/FOXO-associated proteins in the indicated breast cancer cells. **b** and **c** Relative AKT activity and FOXO reporter activity in the indicated cells. Each *bar* represents the mean values ± SD of three independent experiments. *P < 0.05. **d**-**g** Colony formation, anchorage-independent growth, migration and invasion assay revealed the effect of perifosine on the indicated cells. Each *bar* represents the mean values ± SD of three independent experiments. *P < 0.05. *DMSO* dimethyl sulfoxide

GSEA was further performed to identify the pathways involved in TMSB10-mediated breast cancer progression. TMSB10 expression level positively correlated with the AKT-activated gene signatures but negatively correlated with the FOXO(s)-activated gene signatures (Additional file 13: Figure S5a), suggesting that the AKT/FOXO pathway may mediate the pro-tumor effects of TMSB10. On analysis of the TCGA breast cancer datasets, TMSB10 was positively associated with the T308 and S473 phosphorylation levels of AKT, but not with AKT expression level (Additional file 13: Figure S5b). Western blot revealed that upregulating TMSB10 increased, while silencing TMSB10 decreased the phosphorylation levels of AKT, GSK3β, FOXO4 and FOXO3a (Fig. 5a). The analysis of AKT activity revealed that AKT activity was indeed increased in the TMSB10-overexpressing cells and decreased in the TMSB10-silenced cells (Fig. 5b). However, TMSB10 had the opposite effect on the FOXO transcriptional activity (Fig. 5c). These results suggest that the activity of AKT signaling is modulated by TMSB10.

To further determine whether AKT signaling is involved in the pro-tumor role of TMSB10 in breast cancer, we applied the AKT kinase inhibitor perifosine to TMSB10-overexpressing breast cancer cells. As shown in Fig. 5d and e, the colony formation and anchorage-independent growth assays revealed that inhibition of AKT activity decreased the colony and anchorage-independent growth ability of breast cancer cells. Meanwhile, inhibition of AKT kinase activity abrogated the stimulatory effect of TMSB10 on the migration and invasion abilities of breast cancer cells (Fig. 5f and g). Taken together, these findings suggest that TMSB10 promotes tumor progression and metastasis in breast cancer by activating the AKT/FOXO pathway.

### TMSB10 is a valuable serum marker in patients with breast cancer

**Fig. 6:**
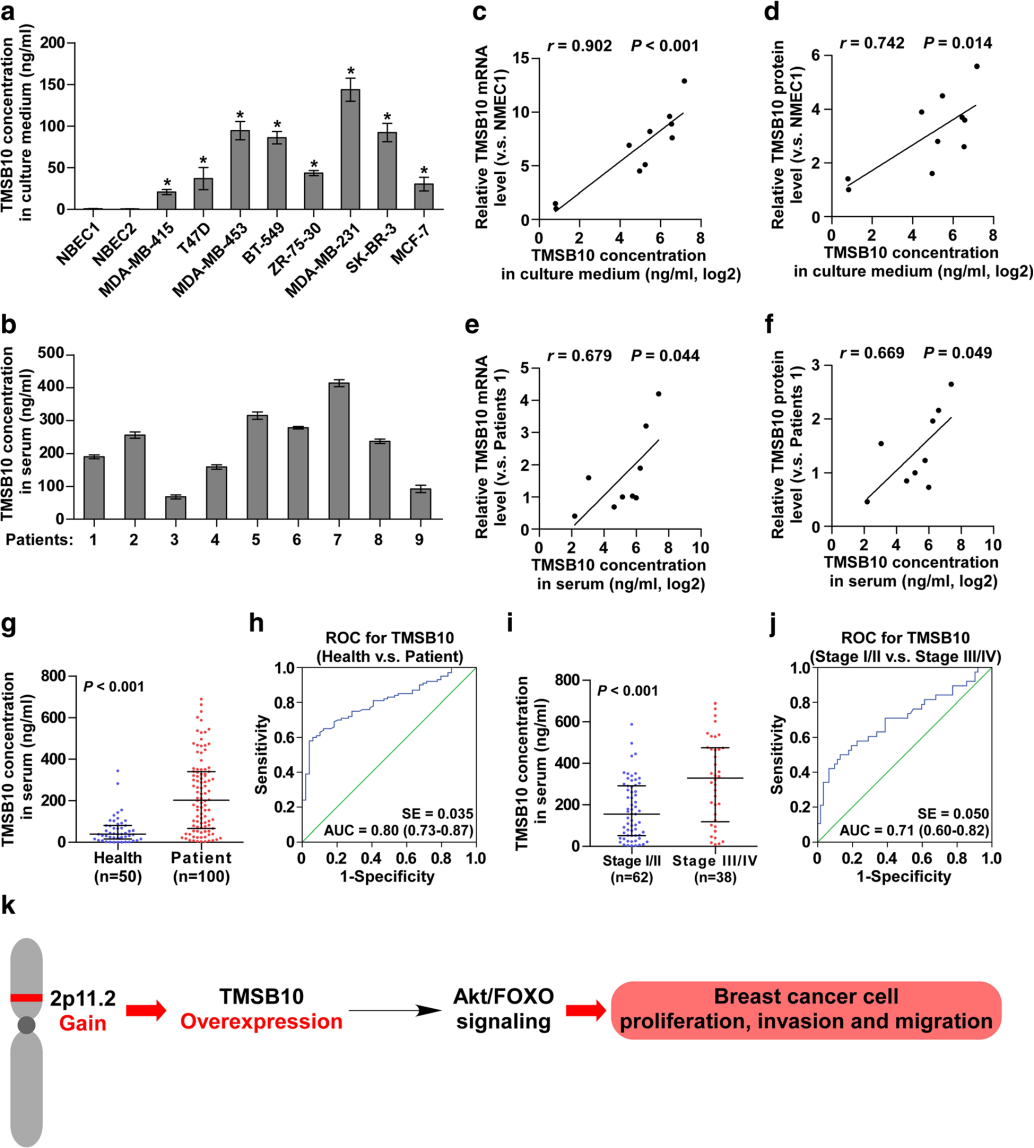
Thymosin beta 10 (*TMSB10*) is a serum marker in patients with breast cancer. **a** TMSB10 expression in the supernatants of NMEC1, NMEC2 and breast cancer cell lines. **b** TMSB10 expression in the serum of nine paired patients with breast cancer. **c**-**f** Correlation between mRNA and protein expression levels of TMSB10 in the separate cells and tissue and the concentration of TMSB10 in their respective supernatants. **g** The concentration of TMSB10 in the serum of patients with breast cancer and healthy serum. **h** Receiver operating characteristic (*ROC*) curve for TMSB10 in healthy individuals and patients with breast cancer. **i** Concentration of TMSB10 in the serum of patients with breast cancer stratified by stage I/II and stage III/IV. **j** ROC curve for TMSB10 in patients with breast cancer at different stages. **k** Hypothetical model of TMSB10 in the regulation of proliferation, migration and invasion in breast cancer cells

Mounting evidence indicates that several members of thymosin, including TMSB10, are secreted proteins both in the physiological and pathological condition [24–28]. Therefore, we examined the protein levels in the supernatants of breast cancer cells using ELISA, and found TMSB10 was highly expressed in breast cancer cells compared with NMECs (Fig. 6a). The previous studies indicated that TMSB4 can be identified in the human serum [29, 30]. So, we further measured the expression levels of TMSB10 in the serum of nine patients with breast cancer and detected TMSB10 at the approximate concentration range of 100–400 ng/ml (Fig. 6b). Moreover, mRNA and protein expression levels of TMSB10 in the separate cell and tissue were consistent with the concentration of TMSB10 in their respective supernatants (Fig. 6c-f).

We further examined the TMSB10 expression in the serum of a large set of patients with breast cancer and found that expression of TMSB10 in the serum was dramatically increased compared with healthy serum (Fig. 6g). Receiver operating characteristic (ROC) analysis of patients with breast cancer gave an area under the curve (AUC) of 0.80 (95% CI = 0.73 to 0.87), Fig. 6h). It is of great interest to find that TMSB10 expression in the serum of patients with breast cancer positively correlated with the clinical stage of breast cancer (Fig. 6i) and on ROC analysis of patients with breast cancer of different stages, the AUC was 0.71 (95% CI = 0.60 to 0.82), Fig. 6j). Therefore, our results indicate that TMSB10 may be identified as a valuable serum biomarker for the diagnosis and clinical staging of breast cancer. Together, these data indicate that TMSB10 plays an important role in tumorigenesis and metastasis in patients with breast cancer (Fig. 6k).

## Discussion

TMSB10 is upregulated in multiple human cancers and upregulation of TMSB10 contributes to cancer cell proliferation, migration and invasion via different mechanisms, and predicts poor survival [13, 15, 31–33]. However, the expression of TMSB10 has also been shown to be downregualted in ovarian cancer tissues and cells and overexpression of TMSB10 diminishes tumor growth and proliferation [34, 35]. These findings indicate that TMSB10 functions as both an oncogenic and tumor-suppressive gene depending on the tumor type. Notably, Santelli and the colleagues demonstrated that overexpression of TMSB10 is a general phenomenon in human carcinogenesis, including that of breast cancer [31]. Furthermore, Verghese et al. report that the intensity of TMSB10 staining positively correlates with the overall grade in breast cancer cells that are characterized by poor differentiation and high mitotic activity, suggesting that TMSB10 may promote the progression of breast cancer by accelerating the cell cycle of breast cancer cells [36]. However, the biological role and clinical significance of TMSB10 in breast cancer remains largely unknown.

Consistently, our results revealed that TMSB10 is elevated in human breast cancer cells and tissues. High expression of TMSB10 correlated with advanced clinicopathological features, poor prognosis and distant metastatic status. Importantly, TMSB10 was significantly elevated in the serum of patients with breast cancer and positively associated with the clinical stages of breast cancer. Our results further revealed that overexpression of TMSB10 promoted, while silencing of TMSB10 inhibited, proliferation, invasion and migration of breast cancer cells by activating the AKT/FOXO signaling pathway in vitro and in vivo. Our finding demonstrates that TMSB10 plays an important role in the progression and metastasis of breast cancer. Intriguingly, Sribenja et al. report that expression of TMSB10 is decreased in metastatic cholangiocarcinoma tissues compared with primary tumors. Silencing TMSB10 significantly increases, while overexpression of TMSB10 reduces, cell migration and invasion of cholangiocarcinoma cells in vitro [37]. Taken together, our results in combination with others indicate that the pro-tumor and anti-tumor effects of TMSB10 on cancer are environmentally and tumor-type dependent.

There is evidence that overexpression of thymosin beta10 results in the increased motility and spread of breast cancer cells as actin-sequestering protein [38]. Consistent with this finding, our results demonstrate that upregulating TMSB10 promotes the invasion and migration of breast cancer cells in vitro and in vivo. So, how might TMSB10 promote tumor metastasis? Emerging studies have revealed that TMSB10 regulates actin dynamics by inhibiting Ras and further disrupting actin polymerization [34, 35]. Cytoskeleton-regulating proteins, including rhodopsin (RHO), CDC42 and ras-related C3 botulinum toxin substrate 1 RAC1 (RAC1), which are members of the RAS family due to high similarity, are implicated in tumor metastasis [39]. Normal epithelial cells are characterized by cell polarity and a well-organized array of specialized cell–cell junctions, where RHO proteins are reported to be involved in the control of epithelial polarity [39]. Furthermore, downregulation of RAC1 leads to a loss in polarity and the loss of epithelial-cell junctions owing to the failure to deposit the extracellular matrix (ECM) component laminin asymmetrically, and establish a more mesenchymal, motile phenotype [40, 41].

Accumulating studies indicate that CDC42 and RAC play important roles in the assembly of actin-rich structures, such as filopodia, which are thought to sense tactic signals and drive the directionality of cell movement, and lamellipodia, which are assembled at the edge of the cells, both of which are demonstrated to be involved in forming integrin-based cell–ECM contacts [42]. These studies indicate that the downregulation or loss of functions of certain cytoskeleton-associated protein might be crucial in the detachment of tumor cells from the ECM and drive the metastasis of tumor cells. Therefore, we speculate that TMSB10 promotes the metastasis of tumor cells probably by inhibiting RAS-related proteins and actin polymerization, leading to the loss of cell–cell junctions, cell polyrization and cell-ECM contacts. However, the specific mechanism is still to be studied further.

Emerging evidence reveals that several proliferation markers, such as Ki67, could predict chemotherapy response in breast cancer because rapidly dividing cells are generally susceptible to chemotherapy. Bertucci et al. analyzed the expression value of Ki67 IHC and mRNA status in node-positive patients with breast cancer treated with adjuvant anthracycline-based chemotherapy in the prospective PACS01 trial, and compared the correlation between Ki67 expression level and histo-clinical variables including disease-free survival (DFS). The patients with high expression of Ki67 exhibited longer disease-free survival compared to patients with low Ki67 expression after chemotherapy, suggesting that patients with high expression of Ki67 have better response to chemotherapy [43]. Furthermore, another study reported that cancer cells with high proliferation rates display pathological complete response to neoadjuvant chemotherapy in breast cancer [44]. These findings indicate that cells with high proliferation respond better to chemotherapy, which significantly improves the survival of patients with cancer.

In this study, TMSB10 was upregulated in breast cancer cell lines and tissues and high expression of TMSB10 indicated poor prognosis in patients with breast cancer. Overexpression of TMSB10 promoted, while silencing TMSB10 inhibited, the proliferation and tumorigenesis of breast cancer cells in vitro and in vivo. These results suggest that TMSB10 promotes the progression of breast cancer by accelerating tumor cell proliferation. Therefore, we wondered whether high expression of TMSB10 can predict chemotherapy response in breast cancer as does Ki67. The analysis of breast cancer datasets from Kaplan-Meier Plotter revealed that patients with high TMSB10 expression had longer disease-free survival compared to patients with low TMSB10 expression after chemotherapy, but this was not seen in patients treated with endocrine therapy or without systemic treatment, because rapidly dividing cells are generally susceptible to chemotherapy.

These results imply that TMSB10 may be used as a potential indicator of chemotherapeutic response, to stratify patients for the treatment of breast cancer. Furthermore, we observed positive correlation between TMSB10 and Ki67 expression in clinical breast cancer tissue samples and tumor tissues formed by the TMSB10-overexpressing cells in vivo. Importantly, TMSB10 can be detected in the serum of patients with breast cancer, indicating that it is clinically more convenient to measure the expression of TMSB10 than Ki67, which will help to predict the chemotherapeutic response of patients with breast cancer. However, further validation is needed in a larger series of studies.

There is still one question pending: why do patients with breast cancer with high TMSB10 expression respond better to chemotherapy than to endocrine therapy? Probably the mechanisms by which chemotherapeutic agents and endocrine therapy kill cancer cells are totally different. Chemotherapy functions by influencing DNA synthesis, which occurs in the S phase of the cell cycle. The accelerated G1-S phase transition and high proliferation rate are important characteristics of cancer cells, which are crucial for cancer cells to be sensitive to chemotherapy. It is worth noting that TMSB10 expression from TCGA analysis was strikingly higher in basal-like and Her2 subtypes of breast cancer, both of which presented more aggressive and malignant phenotypes characterized by high proliferation and high metastatic tendency compared with other subtypes. Furthermore, chemotherapy is the primary therapeutic strategy for basal-like and Her2 subtype of breast cancer. Therefore, we speculate that TMSB10 will exhibit greater applicable values as an indicator for predicting chemotherapeutic response in patients with the basal-like and Her2 subtypes of breast cancer.

Aberrant TMSB10 expression has been implicated in the pathogenesis of human cancer. A study of non-small cell lung cancer (NSCLC) using methylation-specific PCR (MSP) found that TMSB10 promoter was unmethylated in most tumor tissues and became demethylated in 20 (14.4%) of the 139 NSCLCs. However, TMSB10 methylation status was not linked to its overexpression, indicating that hypomethylation may not be a common mechanism underlying TMSB10 overexpression [15]. In this study, we analyzed the methylation array dataset of breast cancer from TCGA and found that there was no obvious discrepancy between tumors and the matched adjacent normal tissue samples in the methylation levels of TMSB10. Collectively, these results indicate that a potent, non-methylation-driven mechanism may underlie the deregulation of TMSB10 expression in breast cancer.

We further analyzed the breast cancer datasets from TCGA and Tumorscape (http://www.broadinstitute.org/tumorscape/) and found that recurrent gains (amplification) were found in approximately 15% of patients with breast cancer. The average expression level of TMSB10 in patients with breast cancer and gains was dramatically higher than those without gains. Frequent amplification has been demonstrated to encode several gene signatures driving cancer growth and plays a crucial role in the progression of cancer [45]. Mounting studies indicate that amplification frequently occurs in many well-known oncogenes, such as ERBB2 and CCND1 [46, 47]. In this study, we examined TMSB10 in our nine paired breast cancer tissue samples using RT-PCR and detected amplification in two of the nine samples. The mRNA expression level of TMSB10 in patients with breast cancer and gains was significantly elevated compared to those without gains. Thus, it is generally believed that recurrent gains (amplification) are associated with high expression of TMSB10 in breast cancer.

## Conclusions

In summary, our results indicate that TMSB10 plays an important role in tumorigenesis and metastasis in patients with breast cancer. The expression level of TMSB10 in the serum of patients with breast cancer is significantly associated with the clinical stages of breast cancer. Furthermore, analysis using Kaplan-Meier Plotter indicates that high expression of TMSB10 predicts better response to chemotherapy in patients with breast cancer. Therefore, detection of TMSB10 in the serum of patients with breast cancer will facilitate the diagnosis and better predict chemotherapy response in patients with breast cancer.

## Additional files

Additional file 1: Table S1. Clinicopathological characteristics of 253 patients with breast cancer for IHC analysis. (PDF 61 kb)
Additional file 2: Table S2. Clinicopathological characteristics of 100 patients with breast cancer for serological detection of TMSB10. (PDF 54 kb)
Additional file 3: Figure S1. TMSB10 is upregulated in breast cancer. **a** TMSB10, PTMA, PTMS, TMSB4X, TMSB15A and TMSB15B expression levels were upregulated to varying degrees in the microarray of breast cancer of the TCGA dataset. **b** Expression of TMSB10 was upregulated in 1099 breast cancer tissue samples compared with 111 normal breast tissue samples in the TCGA profile (**c** and **d**). TMSB10 expression levels in different subtypes of breast cancer from the breast cancer datasets of TCGA and E-GEOD-58135 profiles. Each *bar* represents the median values ± quartile values. **P* < 0.05. **e** Methylation level of TMSB10 in the TCGA breast cancer dataset (**f** and **g**). The percentage of gain in the breast cancer samples from Tumorscape and TCGA. (PDF 305 kb)
Additional file 4: Table S6. Clinicopathological characteristics of patients with breast cancer for analysis in Kaplan-Meier Plotter. (PDF 14 kb)
Additional file 5: Table S7. Clinicopathological characteristics of 1097 patients with breast cancer for analysis in TCGA. (PDF 62 kb)
Additional file 6: Table S8. Primers used in the reactions for real-time RT-PCR. (PDF 9 kb)
Additional file 7: Figure S2. High TMSB10 expression in breast cancer tissue samples correlates with poor patient survival and distant metastasis-free survival. **a** Number of different staining indices of IHC for all 253 patients with breast cancer. **b** and **c** Overall survival and distant metastasis-free survival curves from the TCGA profiles for all 1094 patients with breast cancer stratified by high and low expression of TMSB10. **d**-**f** Overall survival, relapse-free survival and distant metastasis-free survival curves from the Kaplan-Meier Plotter (*BC*) profiles for patients with breast cancer stratified by high and low expression of TMSB10. **g** Kaplan-Meier relapse-free survival curves for all patients with breast cancer stratified by high and low expression of TMSB10 without systemic treatment. **h** Kaplan-Meier relapse-free survival curves for all patients with breast cancer stratified by high and low expression of TMSB10 after endocrine therapy. **i** Kaplan-Meier relapse-free survival curves for all patients with breast cancer stratified by high and low expression of TMSB10 after chemotherapy. (PDF 360 kb)
Additional file 8: Table S3. Relationship between TMSB10 expression and clinicopathological characteristics in 253 patients with breast cancer was tested by chi-square test or Fisher’s test. (PDF 107 kb)
Additional file 9: Table S4. Univariate and multivariate analysis of factors associated with overall survival in 253 patients with breast cancer. (PDF 55 kb)
Additional file 10: Table S5. Univariate and multivariate analysis of factors associated with distant metastasis-free survival in 246 patients with breast cancer. (PDF 55 kb)
Additional file 11: Figure S3. TMSB10 promotes the cell cycle of breast cancer cells. **a** and **b** Real-time PCR and western blot of the indicated breast cancer cells transfected with TMSB10-vector, TMSB10, TMSB10-RNAi-vector, TMSB10-RNAi#1 and TMSB10-RNAi#2. Each *bar* represents the mean values ± SD of three independent experiments. **P* < 0.05. **c** Representative images of sections sliced from the indicated tumors and stained with anti-TMSB10 and anti-Ki67, respectively (*left panel*). Average Ki67 staining score in the indicated tissues (*right panel*). **d** Representative micrographs and quantification of BrdU incorporation in the indicated cells. Each *bar* represents the mean values ± SD of three independent experiments. **P* < 0.05. **e** Real-time PCR analysis of cyclinD1, cyclinE1, CDK6, CDK4, CDKN1A and CDKN1B expression in the indicated cells. Each *bar* represents the mean values ± SD of three independent experiments. **P* < 0.05. **f** Western blot analysis of cyclinD1, cyclinE1, CDK6, CDK4, CDKN1A, CDKN1B and p-Rb expression in the indicated cells. (PDF 354 kb)
Additional file 12: Figure S4. TMSB10 metastasis. **a** The GSEA plot shows that TMSB10 expression positively correlates with metastasis-activated gene signatures. **b** 3D spheroid invasion assay revealed that upregulating TMSB10 increased, while silencing TMSB10 decreased the number of outward projections in the indicated cells. (PDF 167 kb)
Additional file 13: Figure S5. Activation of the Akt/FOXO signaling by TMSB10. **a** The GSEA plot shows that TMSB10 expression positively correlates with Akt-activated gene signatures and negatively correlates with the FOXO3/4-activated gene signatures. **b** TMSB10 expression level is positively associated with the T308 and S473 phosphorylation levels of AKT, but not with AKT level. Each *bar* represents the median values ± quartile. (PDF 231 kb)

## Abbreviations

ANT: Adjacent normal tissue
AUC: Area under the curve
BLI: Bioluminescence imaging
BrdU: Bromodeoxyuridine
CCND1: Cyclin D1
CDC42: Cell division cycle 42
ECM: Extracellular matrix
ELISA: Enzyme-linked immunosorbent assay
ER: Estrogen receptor
ERBB2: Erb-B2 receptor tyrosine kinase 2
FOXO: Forkhead box O
GAPDH: Glyceraldehyde-3-phosphate dehydrogenase
GSEA: Gene set enrichment analysis
H&E: hematoxylin and eosin stain
Her2: Human epidermal growth factor receptor 2
IHC: Immunohistochemical analysis
mRNA: Messenger RNA
NSCLC: Non-small cell lung cancer
PCR: Polymerase chain reaction
PR: Progestrone receptor
PTMA: prothymosin, alpha
PTMS: Parathymosin
RAC1: Ras-related C3 botulinum toxin substrate 1
RHO: Rhodopsin
ROC: receiver operating characteristic curve
ShRNA: Short hairpin RNA
T: Tumor
TCGA: The Cancer Genome Atlas
TMSB10: Thymosin beta 10
