# Supplementary material for: Thymosin beta 10 is a key regulator of tumorigenesis and metastasis and a novel serum marker in breast cancer
Source: Breast Cancer Res. 2017 Feb 8;19:15. doi: 10.1186/s13058-016-0785-2 (PMC5299657; doi:10.1186/s13058-016-0785-2)
Supplement: Additional file 1: Table S1. — Clinicopathological characteristics of 253 patients with breast cancer for IHC analysis. (PDF 61 kb) [file 13058_2016_785_MOESM1_ESM.pdf]

Table S1. The clinicopathological characteristics of 253 patients with breast cancer for IHC analysis were presented.

Parameters		Number of cases	Percentage (%)
Gender	Female	253	100.0
Age (years)	≤50	114	45.1
	>50	139	54.9
Pathological type	IDC	224	88.5
	Other	29	11.5
T classification	T_1	110	43.5
	T_2	108	42.7
	T_3	32	12.6
	T_4	3	1.2
N classification	N_0	132	52.2
	N_1	95	37.5
	N_2	21	8.3
	N_3	5	2.0
M classification	M_0	246	97.2
	M_1	7	2.8
Clinical stage	I	105	41.5
	II	91	36.0
	III	50	19.8
	IV	7	2.8
Pathological grade	G_1	84	33.2
	G_2	90	35.6
	G_3	79	31.2
Survival status	Live	150	59.3
	Dead	103	40.7
Distant metastasis status (Exclude M_1)	Negative	130	52.8
	Positive	116	47.2

IHC status of TMSB10	Low	99	39.1
	High	154	60.9
IHC status of ER	Negative	121	47.8
	Positive	132	52.2
IHC status of PR	Negative	89	35.2
	Positive	164	64.8
IHC status of HER2	Negative	182	71.9
	Positive	71	28.1
IHC status of Ki67	Low	150	59.3
	High	103	40.7

Abbreviation: Invasive ductal carcinoma (IDC), Immunological Histological Chemistry (IHC), Estrogen receptor (ER), Progestrone receptor (PR).
