# Supplementary material for: Thymosin beta 10 is a key regulator of tumorigenesis and metastasis and a novel serum marker in breast cancer
Source: Breast Cancer Res. 2017 Feb 8;19:15. doi: 10.1186/s13058-016-0785-2 (PMC5299657; doi:10.1186/s13058-016-0785-2)
Supplement: Additional file 2: Table S2. — Clinicopathological characteristics of 100 patients with breast cancer for serological detection of TMSB10. (PDF 54 kb) [file 13058_2016_785_MOESM2_ESM.pdf]

Table S2. The clinicopathological characteristics of 100 breast cancer patients for serological detection of TMSB10 were presented.

Parameters		Number of cases	Percentage (%)
Gender	Female	100	100.0
Age (years)	≤50	35	35.0
	>50	65	65.0
Pathological type	IDC	92	92.0
	Other	8	8.0
T classification	T_1	33	33.0
	T_2	36	46.0
	T_3	24	24.0
	T_4	7	7.0
N classification	N_0	29	29.0
	N_1	33	33.0
	N_2	24	24.0
	N_3	14	14.0
M classification	M_0	95	95.0
	M_1	5	5.0
Clinical stage	I	28	28.0
	II	34	34.0
	III	33	33.0
	IV	5	5.0
Pathological grade	G_1	27	27.0
	G_2	35	35.0
	G_3	38	38.0

Abbreviation: Invasive ductal carcinoma (IDC).
