# Supplementary figures and images for: Thymosin beta 10 is a key regulator of tumorigenesis and metastasis and a novel serum marker in breast cancer
Source: Breast Cancer Res. 2017 Feb 8;19:15. doi: 10.1186/s13058-016-0785-2 (PMC5299657; doi:10.1186/s13058-016-0785-2)

Figure S1

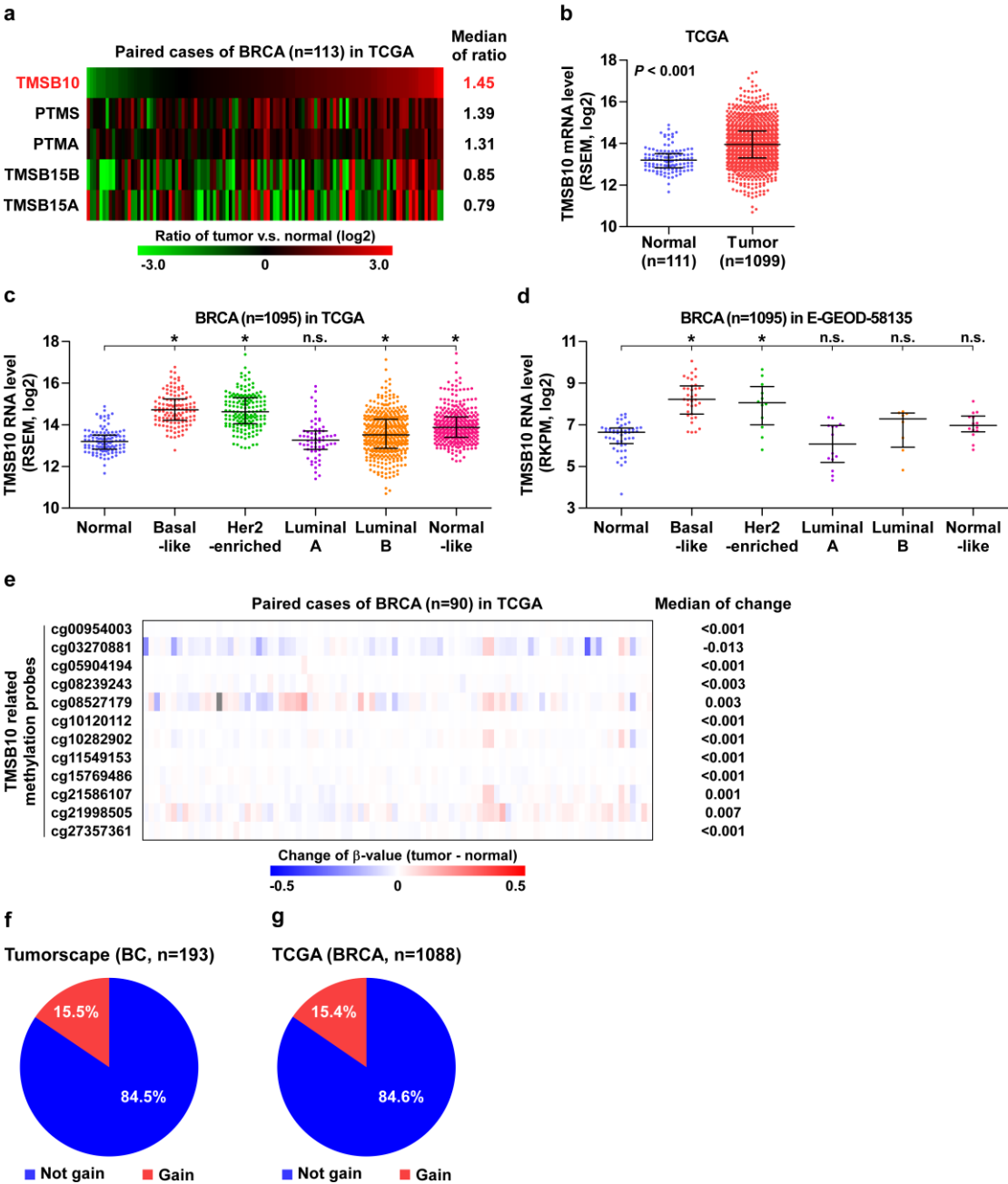

Supplement: Additional file 3: Figure S1. — TMSB10 is upregulated in breast cancer. a TMSB10, PTMA, PTMS, TMSB4X, TMSB15A and TMSB15B expression levels were upregulated to varying degrees in the microarray of breast cancer of the TCGA dataset. b Expression of TMSB10 was upregulated in 1099 breast cancer tissue samples compared with 111 normal breast tissue samples in the TCGA profile (c and d). TMSB10 expression levels in different subtypes of breast cancer from the breast cancer datasets of TCGA and E-GEOD-58135 profiles. Each bar represents the median values ± quartile values. *P < 0.05. e Methylation level of TMSB10 in the TCGA breast cancer dataset (f and g). The percentage of gain in the breast cancer samples from Tumorscape and TCGA. (PDF 305 kb) [file 13058_2016_785_MOESM3_ESM.pdf]

**Figure S2**

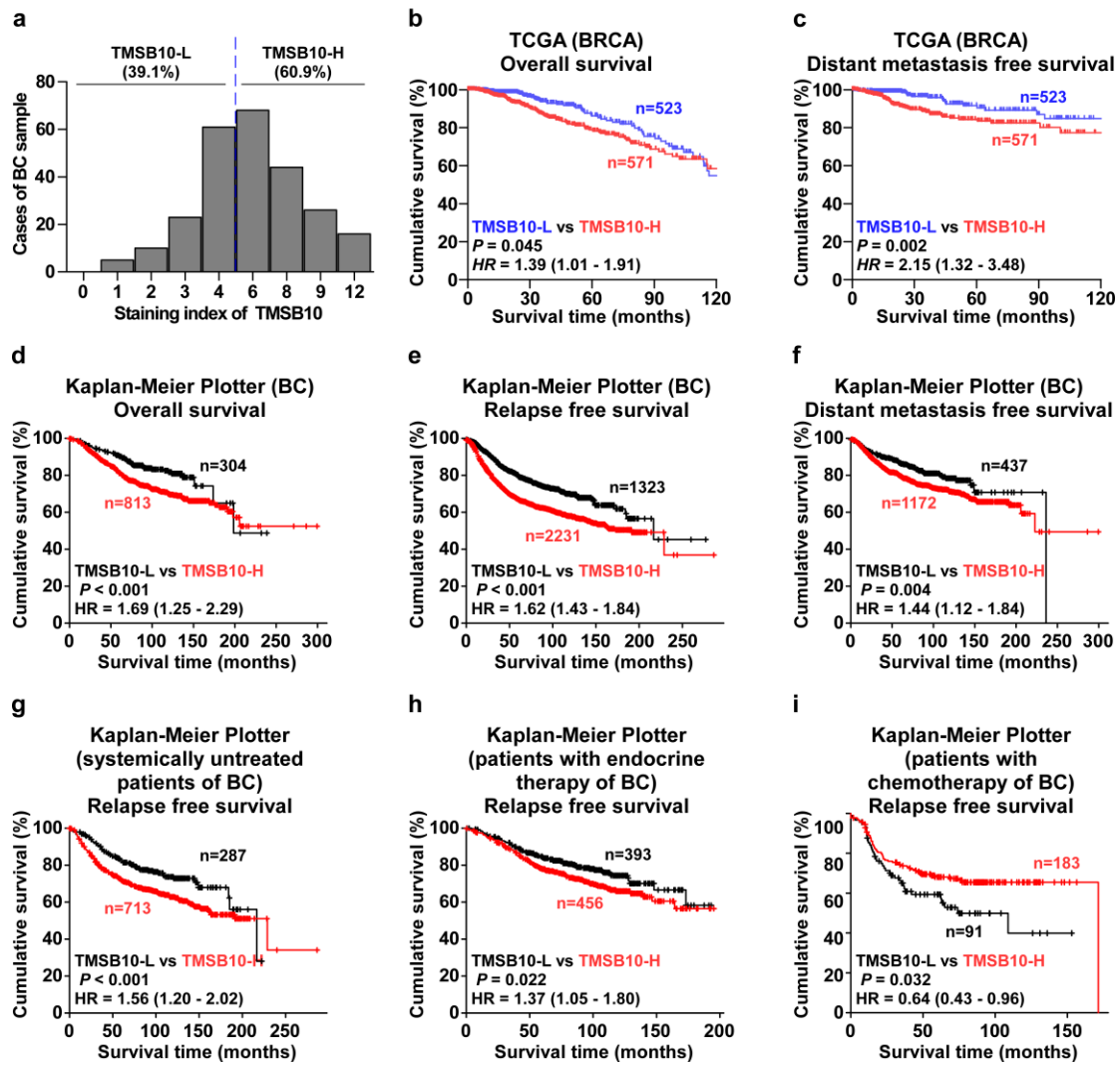

Supplement: Additional file 7: Figure S2. — High TMSB10 expression in breast cancer tissue samples correlates with poor patient survival and distant metastasis-free survival. a Number of different staining indices of IHC for all 253 patients with breast cancer. b and c Overall survival and distant metastasis-free survival curves from the TCGA profiles for all 1094 patients with breast cancer stratified by high and low expression of TMSB10. d-f Overall survival, relapse-free survival and distant metastasis-free survival curves from the Kaplan-Meier Plotter (BC) profiles for patients with breast cancer stratified by high and low expression of TMSB10. g Kaplan-Meier relapse-free survival curves for all patients with breast cancer stratified by high and low expression of TMSB10 without systemic treatment. h Kaplan-Meier relapse-free survival curves for all patients with breast cancer stratified by high and low expression of TMSB10 after endocrine therapy. i Kaplan-Meier relapse-free survival curves for all patients with breast cancer stratified by high and low expression of TMSB10 after chemotherapy. (PDF 360 kb) [file 13058_2016_785_MOESM7_ESM.pdf]

**Figure S3**

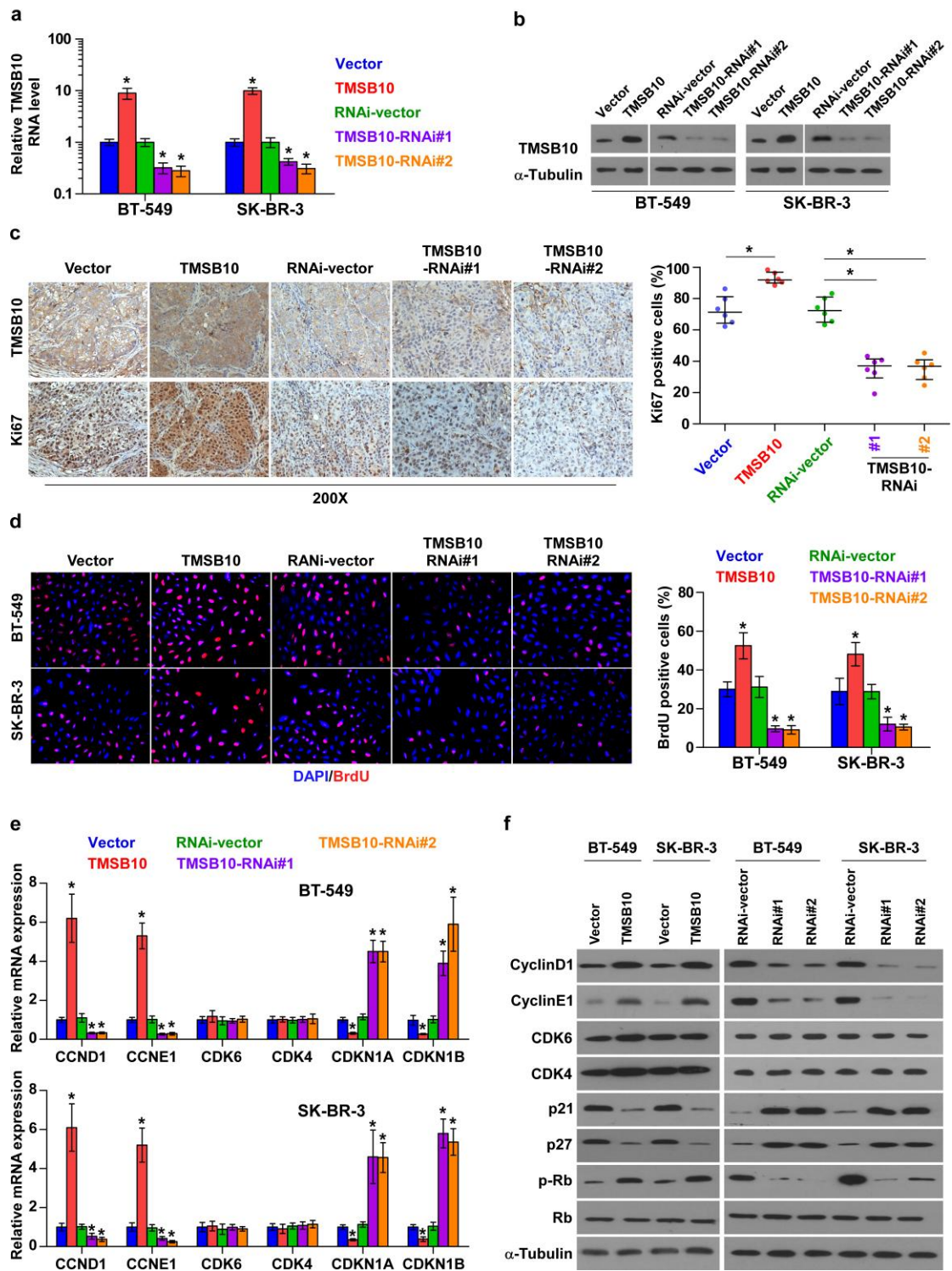

Supplement: Additional file 11: Figure S3. — TMSB10 promotes the cell cycle of breast cancer cells. a and b Real-time PCR and western blot of the indicated breast cancer cells transfected with TMSB10-vector, TMSB10, TMSB10-RNAi-vector, TMSB10-RNAi#1 and TMSB10-RNAi#2. Each bar represents the mean values ± SD of three independent experiments. *P < 0.05. c Representative images of sections sliced from the indicated tumors and stained with anti-TMSB10 and anti-Ki67, respectively (left panel). Average Ki67 staining score in the indicated tissues (right panel). d Representative micrographs and quantification of BrdU incorporation in the indicated cells. Each bar represents the mean values ± SD of three independent experiments. *P < 0.05. e Real-time PCR analysis of cyclinD1, cyclinE1, CDK6, CDK4, CDKN1A and CDKN1B expression in the indicated cells. Each bar represents the mean values ± SD of three independent experiments. *P < 0.05. f Western blot analysis of cyclinD1, cyclinE1, CDK6, CDK4, CDKN1A, CDKN1B and p-Rb expression in the indicated cells. (PDF 354 kb) [file 13058_2016_785_MOESM11_ESM.pdf]

Figure S4

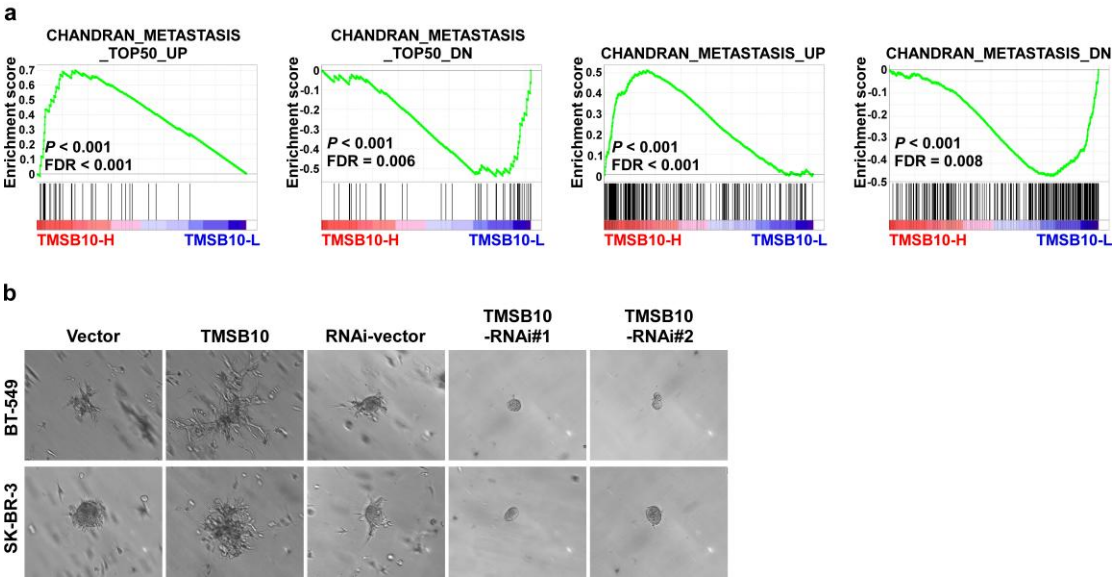

Supplement: Additional file 12: Figure S4. — TMSB10 metastasis. a The GSEA plot shows that TMSB10 expression positively correlates with metastasis-activated gene signatures. b 3D spheroid invasion assay revealed that upregulating TMSB10 increased, while silencing TMSB10 decreased the number of outward projections in the indicated cells. (PDF 167 kb) [file 13058_2016_785_MOESM12_ESM.pdf]

Figure S5

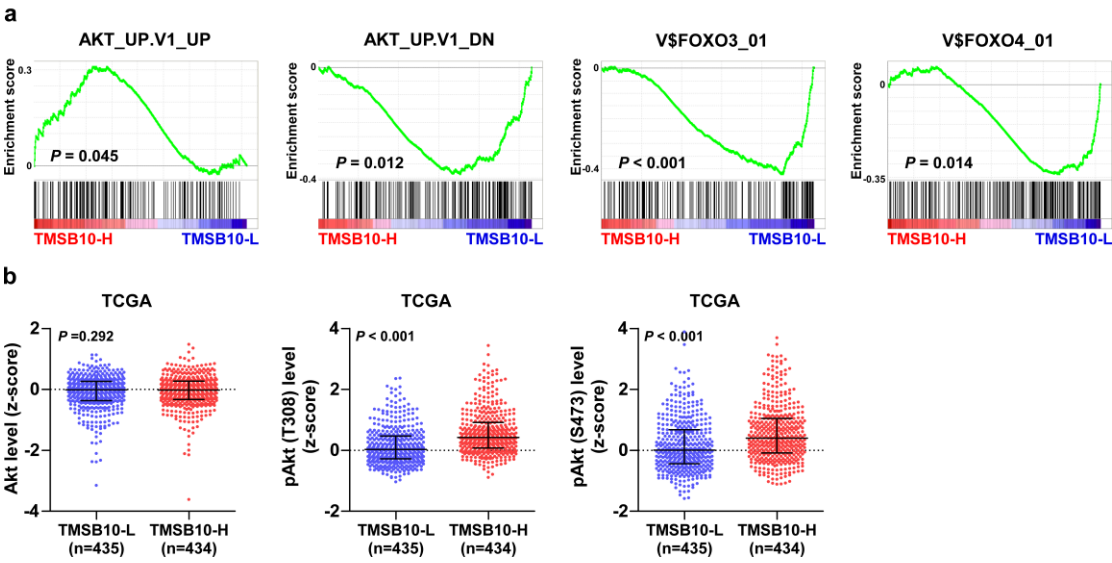

Supplement: Additional file 13: Figure S5. — Activation of the Akt/FOXO signaling by TMSB10. a The GSEA plot shows that TMSB10 expression positively correlates with Akt-activated gene signatures and negatively correlates with the FOXO3/4-activated gene signatures. b TMSB10 expression level is positively associated with the T308 and S473 phosphorylation levels of AKT, but not with AKT level. Each bar represents the median values ± quartile. (PDF 231 kb) [file 13058_2016_785_MOESM13_ESM.pdf]
