# Supplementary material for: Thymosin beta 10 is a key regulator of tumorigenesis and metastasis and a novel serum marker in breast cancer
Source: Breast Cancer Res. 2017 Feb 8;19:15. doi: 10.1186/s13058-016-0785-2 (PMC5299657; doi:10.1186/s13058-016-0785-2)
Supplement: Additional file 4: Table S6. — Clinicopathological characteristics of patients with breast cancer for analysis in Kaplan-Meier Plotter. (PDF 14 kb) [file 13058_2016_785_MOESM4_ESM.pdf]

Table S6. The clinicopathological characteristics of patients in Kaplan-Meier Plotter with breast cancer for analysis were presented.

Parameters		Number of cases
ER status (n = 3779)	Positive	2565
	Negative	1214
PR status (n = 1982)	Positive	954
	Negative	1028
HER2 status (n = 1872)	Positive	416
	Negative	1456
Lymph node status (n = 3720)	Positive	1459
	Negative	2259
Grade (n = 2545)	I	378
	II	1077
	III	1090
Intrinsic subtype (n = 5143)	Basal	879
	Luminal A	2504
	Luminal B	1425
	HER2+	335
TP53 status (n = 595)	Mutated	232
	Wild type	363
Pietenpol subtype (n = 1246)	Basal-like 1	239
	Basal-like 2	97
	Immunomodulatory	290
	Mesenchymal	229
	Mesenchymal stem-like	115
	Luminal androgen receptor	276
With systemically therapy (n = 3899)	Include	2884
	Exclude	1015
With endocrine therapy (n = 3099)	Include	1381
	Exclude	1718

	Tamoxifen only	1043
With Chemotherapy (n = 3268)	Include	678
	Exclude	2590

Abbreviation: Estrogen receptor (ER), Progestrone receptor (PR), Human epidermal growth factor receptor 2 (HER2).
