# Supplementary material for: Thymosin beta 10 is a key regulator of tumorigenesis and metastasis and a novel serum marker in breast cancer
Source: Breast Cancer Res. 2017 Feb 8;19:15. doi: 10.1186/s13058-016-0785-2 (PMC5299657; doi:10.1186/s13058-016-0785-2)
Supplement: Additional file 5: Table S7. — Clinicopathological characteristics of 1097 patients with breast cancer for analysis in TCGA. (PDF 62 kb) [file 13058_2016_785_MOESM5_ESM.pdf]

Table S7. The clinicopathological characteristics of 1097 patients in TCGA with breast cancer for analysis were presented.

Parameters		Number of cases	Percentage (%)
Gender	Female	1085	98.9
	Male	12	1.1
Age (years)	≤50	766	69.8
	>50	331	30.2
Pathological type	IDC	784	71.5
	Other	312	28.4
	NA	1	0.1
T classification	T_1	281	25.6
	T_2	635	57.9
	T_3	138	12.6
	T_4	40	3.6
	TX	3	0.3
N classification	N_0	516	47.0
	N_1	364	33.2
	N_2	120	10.9
	N_3	77	7.0
	NX	20	2.0
M classification	M_0	912	83.1
	M_1	22	2.0
	MX	163	14.9
Clinical stage	I	183	16.7
	II	621	56.6
	III	249	22.7
	IV	20	1.8
	NA	24	2.2
Survival status	Live	945	86.1

	Dead	152	13.9
	Negative	755	68.8
Relapse status	Positive	157	14.3
	NA	185	16.9
	Negative	238	21.7
IHC status of ER	Positive	808	73.7
	NA	51	4.6
	Negative	344	31.4
IHC status of PR	Positive	699	63.7
	NA	54	4.9
	Negative	564	51.4
IHC status of HER2	Positive	164	14.9
	NA	369	33.6

Abbreviation: Invasive ductal carcinoma (IDC), Immunological Histological Chemistry (IHC), Estrogen receptor (ER), Progestrone receptor (PR), Human epidermal growth factor receptor 2 (HER2), Not Available information (NA).
