# Supplementary material for: Thymosin beta 10 is a key regulator of tumorigenesis and metastasis and a novel serum marker in breast cancer
Source: Breast Cancer Res. 2017 Feb 8;19:15. doi: 10.1186/s13058-016-0785-2 (PMC5299657; doi:10.1186/s13058-016-0785-2)
Supplement: Additional file 6: Table S8. — Primers used in the reactions for real-time RT-PCR. (PDF 9 kb) [file 13058_2016_785_MOESM6_ESM.pdf]

Table S8. A list of primers used in the reactions for real-time RT-PCR.

Gene name		Sequence
TMSB10	forward	5'-CTTATCGAAGCTGGCGATTT-3'
	reverse	5'-AGTGGGAGCACCAGGATCT-3'
CCND1	forward	5'-AACTACCTGGACCGCTTCCT-3'
	reverse	5'-CCACTTGAGCTTG TTCACCA-3'
CCNE1	forward	5'-TCTTTGTCAGGTGTGGGGA-3'
	reverse	5'-GAAATGGCCAAAATCGACAG-3'
CDK6	forward	5'-TGTCTG TTCGTGACACTGTGC-3'
	reverse	5'-ATGCCGCTCTCCACCAT-3'
CDK4	forward	5'-GTCGGCTTCAGAGTTTCC AC-3'
	reverse	5'-TGCAGTCCACATATGCAACA-3'
CDKN1A	forward	5'-GTCCACTGGGCCGAAGAG-3'
	reverse	5'-TGCGTTCACAGGTGTTTCTG-3'
CDKN1B	forward	5'-TTCATCAAGCAGT GATGTATCTGA-3'
	reverse	5'-AAGAAGCCTGGCCTCAGAAG-3'
GAPDH	forward	5'-AC CACAGTCCATGCCATCAC-3'
	reverse	5'-TCCACCACCCTG TTGCTGTA-3'
