# Supplementary material for: Thymosin beta 10 is a key regulator of tumorigenesis and metastasis and a novel serum marker in breast cancer
Source: Breast Cancer Res. 2017 Feb 8;19:15. doi: 10.1186/s13058-016-0785-2 (PMC5299657; doi:10.1186/s13058-016-0785-2)
Supplement: Additional file 8: Table S3. — Relationship between TMSB10 expression and clinicopathological characteristics in 253 patients with breast cancer was tested by chi-square test or Fisher’s test. (PDF 107 kb) [file 13058_2016_785_MOESM8_ESM.pdf]

Table S3. The relationship between TMSB10 expression and clinicopathological characteristics in 253 patients with breast cancer was tested by Chi-square test or Fisher's test.

Parameters	Number of cases	TMSB10 expression		*P* values
		Low	High	
Age (years)				
≤50	114	47	67	0.536
>50	139	52	87	
Pathological type				
IDC	224	91	133	0.176
Other	29	8	21	
T stage				
T_1	110	59	51	<0.001*
$T_2 – T_4$	143	40	103	
N stage				
N_0	132	76	56	<0.001*
$N_1 – N_3$	121	23	98	
M stage				
M_0	246	99	147	0.045*
M_1	7	0	7	
Clinical stage				
I – II	196	92	104	<0.001*
III – IV	57	7	50	
Pathological grade				
$G_1 – G_2$	174	81	93	<0.001*
G_3	79	18	61	
IHC status of ER				
Negative	121	34	87	<0.001*
Positive	132	65	67	
IHC status of PR				
Negative	89	26	63	0.017*
Positive	164	73	91	
IHC status of HER2				
Negative	182	77	105	0.097
Positive	71	22	49	
IHC status of Ki67				
Low	150	70	80	0.003*
High	103	29	74	
