# Supplementary material for: Thymosin beta 10 is a key regulator of tumorigenesis and metastasis and a novel serum marker in breast cancer
Source: Breast Cancer Res. 2017 Feb 8;19:15. doi: 10.1186/s13058-016-0785-2 (PMC5299657; doi:10.1186/s13058-016-0785-2)
Supplement: Additional file 9: Table S4. — Univariate and multivariate analysis of factors associated with overall survival in 253 patients with breast cancer. (PDF 55 kb) [file 13058_2016_785_MOESM9_ESM.pdf]

Table S4. Univariate and multivariate analysis of factors associated with overall survival in 253 breast cancer patients.

Characteristics	Univariate analysis		Multivariate analysis	
	HR (95% CI)	*P* values	HR (95% CI)	*P* values
Age (>50 years)	1.27 (0.85-1.87)	0.242	1.28 (0.84-1.93)	0.251
Pathological type (IDC)	1.44 (0.83-2.48)	0.196	0.82 (0.46-1.46)	0.496
T stage (T_2 – T_4)	4.03 (2.49-6.52)	<0.001*	1.59 (0.89-2.84)	0.116
N stage (N_1 – N_3)	6.06 (3.77-9.73)	<0.001*	2.60 (1.45-4.67)	<0.001*
M stage (M_1)	5.88 (2.70-12.8)	<0.001*	1.76 (0.72-4.32)	0.214
Clinical stage (III – IV)	6.03 (4.06-8.95)	<0.001*	1.50 (0.88-2.56)	0.136
Histologic grade (G_3)	3.84 (2.59-5.68)	<0.001*	1.96 (1.24-3.09)	0.004
Status of ER (Positive)	0.68 (0.46-1.00)	0.048*	0.99 (0.63-1.57)	0.976
Status of PR (Positive)	0.54 (0.37-0.80)	0.002	0.69 (0.44-1.07)	0.096
Status of HER2 (Positive)	1.81 (1.22-2.70)	0.003*	1.75 (1.15-2.68)	0.010
Status of TMSB10 (High)	4.62 (2.75-7.79)	<0.001*	2.10 (1.20-3.67)	0.010
Status of Ki67 (High)	2.36 (1.60-3.48)	<0.001*	1.67 (1.11-2.52)	0.014

HR, hazard ratio; CI, confidence interval.
