# Supplementary material for: Thymosin beta 10 is a key regulator of tumorigenesis and metastasis and a novel serum marker in breast cancer
Source: Breast Cancer Res. 2017 Feb 8;19:15. doi: 10.1186/s13058-016-0785-2 (PMC5299657; doi:10.1186/s13058-016-0785-2)
Supplement: Additional file 10: Table S5. — Univariate and multivariate analysis of factors associated with distant metastasis-free survival in 246 patients with breast cancer. (PDF 55 kb) [file 13058_2016_785_MOESM10_ESM.pdf]

Table S5. Univariate and multivariate analysis of factors associated with distant metastasis-free survival in 246 breast cancer patients.

Characteristics	Univariate analysis		Multivariate analysis	
	HR (95% CI)	*P* values	HR (95% CI)	*P* values
Age (>50 years)	1.00 (0.69-1.44)	0.996	1.04 (0.71-1.51)	0.861
Pathological type (IDC)	1.54 (0.92-2.58)	0.100	0.88 (0.51-1.52)	0.657
T stage (T_2 – T_4)	3.37 (2.21-5.14)	<0.001*	1.72 (1.05-2.81)	0.032*
N stage (N_1 – N_3)	4.22 (2.83-6.30)	<0.001*	2.07 (1.27-3.39)	0.004*
M stage (M_1)	-	-	-	-
Clinical stage (III – IV)	4.50 (3.05-6.63)	<0.001*	1.34 (0.82-2.21)	0.248
Histologic grade (G_3)	1.73 (1.14-2.64)	0.011*	1.73 (1.14-2.64)	0.011*
Status of ER (Positive)	0.74 (0.51-1.06)	0.102	1.12 (0.73-1.72)	0.591
Status of PR (Positive)	0.49 (0.34-0.71)	<0.001*	0.59 (0.39-0.91)	0.016*
Status of HER2 (Positive)	1.80 (1.23-2.62)	0.002*	1.72 (1.15-2.55)	0.008*
Status of TMSB10 (High)	3.89 (2.48-6.11)	<0.001*	2.07 (1.27-3.35)	0.003*
Status of Ki67 (High)	2.06 (1.43-2.97)	<0.001*	1.47 (1.00-2.16)	0.053

HR, hazard ratio; CI, confidence interval.
